# Very-low-carbohydrate diets and preservation of muscle mass

**DOI:** 10.1186/1743-7075-3-9

**Published:** 2006-01-31

**Authors:** Anssi H Manninen

**Affiliations:** 1 Advanced Research Press, Inc., Setauket, NY, USA

## Background

I would like to compliment Noakes et al. on their well-controlled study comparing effects of different diets on body composition and cardiovascular risk [1]. The authors suggested that a very-low-carbohydrate diet (VLCARB) may not be associated with protein-sparing, because their dual-energy X-ray absorptiometry (DEXA) data indicated that both VLCARB and very-low-fat diet resulted in significantly more loss of lean mass than the high-unsaturated fat diet. It should be noted, however, that DEXA provides a measure of lean soft tissue (LST), and the original notion that LST hydration is constant is not correct. Rather, LST hydration varies as a function of extra- and intracellular water distribution [16]. I feel it is *very *unlikely that the VLCARB group catabolized more muscle protein than the high-unsatured fat diet group. This commentary provides some basic information on metabolic adaptations that lead to sparing of muscle protein during a VLCARB, and reviews studies examining the effects of VLCARB interventions on body composition.

## Metabolic adaptations in VLCARB

It is frequently claimed that a VLCARB sets the stage for a significant loss of muscle mass as the body recruits amino acids from muscle protein to maintain blood glucose via gluconeogenesis. It is true that animals share the metabolic deficiency of the total (or almost total) inability to convert fatty acids to glucose [18]. Thus, the primary source for a substrate for gluconeogenesis is amino acid, with some help from glycerol from fat tissue triglycerides. However, when the rate of mobilization of fatty acids from fat tissue is accelerated, as, for example, during a VLCARB, the liver produces ketone bodies. The liver cannot utilize ketone bodies and thus, they flow from the liver to extra-hepatic tissues (e.g., brain, muscle) for use as a fuel. Simply stated, ketone body metabolism by the brain displaces glucose utilization and thus spares muscle mass. In other words, the brain derives energy from storage fat during a VLCARB.

Glycolytic cells and tissues (e.g., erythrocytes, renal medulla) will still need some glucose, because they do not have aerobic oxidative capacity and thus cannot use ketone bodies. However, glycolysis in these tissues leads to the release of lactate that is returned to the liver and then reconverted into glucose (the Cori cycle). Energy for this process comes from the increased oxidation of fatty acids in the liver. Thus, glycolytic tissues indirectly also run on energy derived from the fat stores.

The hormonal changes associated with a VLCARB include a reduction in the circulating levels of insulin along with increased levels of glucagon. Insulin has many actions, the most well-known of which is stimulation of glucose and amino acid uptake from the blood to various tissues. This is coupled with stimulation of anabolic processes such as protein, glycogen and fat synthesis. Glucagon has opposing effects, causing the release of glucose from glycogen and stimulation of gluconeogenesis and fat mobilization. Thus, the net stimulus would seem to be for increasing muscle protein breakdown. However, a number of studies indicate that a VLCARB results in body composition changes that favour loss of fat mass and preservation in muscle mass.

## A review of studies

To my knowledge, Benoit et al. published the first systematic study of the effect of a VLCARB on composition of weight loss [2]. They reported that when a 1,000-kcal VLCARB (10 g of carbohydrates/day) was fed for 10 days, seven male subjects lost an average of 600 g/day, of which 97% was fat. As pointed out by Grande [11], however, the energy value of tissue loss reported by Benoit et al. calculates out to be about 7,000 kcal/day, a highly improbable level of energy expenditure. In a study by Yang and Van Itallie [20], effects of starvation, an 800-kcal mixed diet and an 800-kcal VLCARB on the composition of weight lost were determined in each of six obese subjects during three 10-day periods. The results indicated that composition of weight lost during the VLCARB and the mixed diet was water 61.2, fat 35.0, protein 3.8, and water 37.1, fat 59.5, protein 3.4 percent, respectively. Thus, the authors concluded that, over a 10-day period, the energy value of body constituents lost during adherence to an 800-kcal is minimally affected by diet composition. *Because of metabolic adaptations to prolonged changes in diet composition, the results of such short-term studies cannot be applied to longer-term situations*. Young et al. compared three diets containing the same amounts of calories (1,800 kcal/day) and protein (115 g/day) but differing in carbohydrate content [3]. After nine weeks on the 30-g, 60-g and 104-g carbohydrate diets, weight loss was 16.2, 12.8 and 11.9 kg and fat accounted for 95, 84, and 75% of the weight loss, respectively. Importantly, underwater weighing was used to determine body composition. Although these results should be interpreted cautiously given the low number of subjects, this study strongly suggests that a VLCARB promotes fat loss while preserving muscle mass, supporting the notion that "a calorie is not a calorie" [23-25]. Phinney et al. reported that subjects lost 0.7 kg in the first week of the eucaloric VLCARB, after which their weight remained stable [15]. Thus, they observed a reduction in glycogen stores, but excellent preservation of muscle protein.

More recently, Willi et al. examined the efficacy and metabolic impact of a VLCARB in the treatment of morbidly obese adolescents [4]. Six adolescents weighing an average of 147.8 kg consumed the VLCARB (25 g of carbohydrate/day) for 8 weeks. The results indicated that the weight loss with VLCARB is rapid, consistent, and almost exclusively from body fat stores. Changes in lean body mass, as estimated from DEXA *and *urinary creatinine, were not significant over the term of treatment. Bioelectrical impedance measurements reflected a greater loss of lean body mass, but changes in total body fluid and electrolyte content, as a result of dietary ketosis, may complicate these measurements.

Volek et al. investigated the effects of a six-week VLCARB on body composition in healthy normal-weight men [5]. Twelve subjects switched from their habitual diet (48% carbohydrates) to a VLCARB (8% percent carbohydrates) for six weeks and eight men served as controls, consuming their normal diet. Although subjects were encouraged to consume adequate dietary energy to maintain body mass during the intervention, the results revealed that fat mass was significantly decreased (-3.4 kg) and lean body mass significantly *increased *(+1.1 kg) at week six (as measured by DEXA). There were no significant changes in composition in the control group. The authors concluded that a VLCARB resulted in a significant reduction in fat mass and an accompanying increase in lean body mass in normal-weight men. In other words, the *entire *loss in bodyweight was from body fat. A subsequent study by Volek et al. using a VLCARB during energy-restriction noted a greater decrease in lean body mass in men who consumed a VLCARB than in men won consumed a high-carbohydrate/low-fat diet. However, resting energy expenditure was maintained in men consuming the VLCARB but decreased on the high-carbohydrate/low-fat diet, strongly suggesting that the VLCARB group did not lose muscle mass.

Vazquez and Adibi reported that proteolysis, as measured by leucine turnover rate and urinary excretion of 3-methylhistidine, was not significantly different between isocaloric VLCARB and non-ketogenic diet [17]. However, this study is not relevant to "normal" weight loss diets, because their subjects consumed only 600 kcal and 8 g of nitrogen per day. Such a semi-starvation diet will lead to increased muscle protein catabolism no matter what the subjects eat.

The perception that the VLCARB leads to progressive loss of muscle protein apparently comes from the poorly controlled "Turkey Study" published in the *New England Journal of Medicine *in 1980 [12]. The authors of this study reported that the protein-only diet subjects were losing nitrogen yet gaining potassium. As pointed out by Phinney [13,14], however, potassium and nitrogen losses are closely related, as they are *both *contained in lean tissue. This anomaly occurred because the authors assumed the potassium intake of their subjects was based upon handbook values for *raw *turkey, but half of this potassium was being discarded in the unconsumed broth. Deprived of potassium, these subjects were unable to benefit from dietary protein and thus lost muscle mass [14].

## How is the preservation of muscle mass brought about during a VLCARB?

There are at least four possible mechanisms:

### Adrenergic stimulation

The increase in adrenaline may be involved. Low blood sugar is a potent stimulus to adrenaline secretion and it is now clear that skeletal muscle protein mass is also regulated by adrenergic influences. For example, Kadowaki et al. demonstrated that adrenaline directly inhibits proteolysis of skeletal muscle [6].

### Ketone bodies

As noted above, the liver produces ketone bodies during a VLCARB and they flow from the liver to extra-hepatic tissues (e.g., brain, muscle) for use as a fuel. In addition, ketone bodies exert a restraining influence on muscle protein breakdown. If the muscle is plentifully supplied with other substrates for oxidation (such as fatty acids and ketone bodies, in this case), then the oxidation of muscle protein-derived amino acids is suppressed. Nair et al. reported that beta-hydroxybutyrate (beta-OHB, a major ketone body) decreases leucine oxidation and promotes protein synthesis in humans [7]. Although blood concentrations of beta-OHB in their subjects during the infusion of beta-OHB were much lower than concentrations observed in humans during fasting, leucine incorporation into skeletal muscle showed a significant increase (5 to 17%).

### Growth hormone (GH)

GH has a major role in regulating growth and development. GH is a protein anabolic hormone and it stimulates muscle protein synthesis. As low blood sugar increases GH secretions, one could speculate that a VLCARB increases GH levels. However, Harber et al. reported that GH secretion was unchanged with 7-day VLCARB/high-protein diet [8]. Interestingly, they also observed that skeletal muscle expression of IGF-I mRNA increased about 2-fold. A plausible explanation for the increased expression of IGF-I in muscle is the increased availability of dietary protein.

### Dietary protein

A VLCARB is almost always relatively high in protein. There is evidence that high protein intake increases protein synthesis by increasing systemic amino acid availability [21], which is a potent stimulus of muscle protein synthesis [22]. During weight loss, higher protein intake reduces loss of muscle mass and increases loss of body fat [9]. It has been proposed that the branched-chain amino acid leucine interacts with the insulin signaling pathway to stimulate downstream control of protein synthesis, resulting in maintenance of muscle mass during periods of restricted energy intake [10]. A recent study by Harber et al. reported that a VLCARB/high-protein diet increases skeletal muscle protein synthesis despite a dramatic reduction in insulin levels [8].

## Conclusion

Although more long-term studies are needed before a firm conclusion can be drawn, it appears, from most literature studied, that a VLCARB is, if anything, *protective *against muscle protein catabolism during energy restriction, provided that it contains adequate amounts of protein.

## Competing interests

The author(s) declare that they have no competing interests.
